# Comparison of the Clinical Spectrum of Juvenile- and Adult-Onset Huntington Disease: A National Cohort and Enroll-HD Observational Study

**DOI:** 10.1212/WNL.0000000000213525

**Published:** 2025-04-22

**Authors:** Hannah S. Bakels, Kasper F. van der Zwaan, Erik Van Zwet, Robert Reijntjes, Gregory P. Sprenger, Thijs A. Knecht, Raymund A.C. Roos, Susanne T. de Bot

**Affiliations:** 1 Department of Neurology, Leiden University Medical Center, the Netherlands; and; 2 Department of Biomedical Data Sciences, Leiden University Medical Center, the Netherlands.

## Abstract

**Background and Objectives:**

Differences in clinical characteristics between juvenile-onset Huntington disease (JHD) and adult-onset HD (AHD) are hypothesized but not directly compared. This study compares clinical characteristics occurrence and severity across age-at-onset (AO) subtypes.

**Methods:**

Using the national juvenile-onset HD patient cohort and the international Enroll-HD registry (NCT01574053), we compared childhood-onset JHD (cJHD; AO 0–10), adolescent-onset JHD (aJHD; AO 11–20), and adult-onset HD (AHD; AO 21–65) on proportions of clinical characteristics at onset and psychiatric characteristics in pooled datasets. Kruskal-Wallis test was applied to UHDRS-Total Motor Score (UHDRS-TMS) items of the Enroll-HD dataset to compare the severity of motor disease characteristics 6–10 years after onset.

**Results:**

The combined datasets provided data from 46 patients with cJHD (mean AO 6.70, 45% female), 243 patients with aJHD (mean AO 16.70, 46% female), and 9,504 patients with AHD (mean AO 44.70, 51% female). At onset, neurocognitive symptoms occurred in 47.50% of patients with cJHD (n = 46; 95% CI 31.80%–63.70%), significantly more often compared with 24.88% of patients with aJHD (n = 209; 19.30%–31.40%) and 15.02% of those with AHD (n = 8,177; 14.30%–15.80%). Psychiatric symptoms occurred in 47.12% of patients with aJHD (95% CI 40.20%–54.10%), significantly more compared with 31.04% of patients with AHD (30.10%–32.00%). Throughout the disease, aggressive behavior occurred in 73.91% of patients with cJHD (n = 46; 95% CI 58.60%–85.20%) and 55.88% of those with aJHD (n = 238; 49.30%–62.30%), significantly more compared with 40.65% of patients with AHD (n = 9,501; 39.70%–41.70%). Psychosis occurred in 23.53% of patients with aJHD (95% CI 18.40%–29.50%), significantly more compared with 12.77% of those with AHD (12.10%–13.50%). The Kruskal-Wallis test revealed significantly higher median UHDRS-TMS scores in one or both JHD subtypes compared with AHD for dysarthria (AHD: n = 4,163, median 1.00, interquartile range (IQR) 0.70; cJHD: n = 12, 2.20, 2.00, *p =* 0.039; aJHD: n = 93, 1.00, 1.00, *p* = 0.031), parkinsonism (AHD: n = 4,158, median 6.00, IQR 4.70; cJHD: n = 12, 11.00, 9.40, *p* = 0.008; aJHD: n = 93, 8.50, 6.80, *p* < 0.001), and dystonia (AHD: n = 4,161, median 2.00, IQR 5.20; cJHD: n = 12, 6.50, 8.20, *p* = 0.141; aJHD: n = 93, 4.00, 7.20, *p =* 0.015) and significantly lower median scores for chorea (AHD: n = 4,163, median 9.20, IQR 7.00; cJHD: n = 12, 5.00, 4.20, *p* = <0.001; aJHD: n = 93, 6.30, 9.50, *p* < 0.001).

**Discussion:**

This study highlights distinct clinical patterns in JHD subtypes compared with AHD. Stratification by age at onset–defined HD subtypes is needed in future studies.

## Introduction

Huntington disease (HD) is an autosomal dominant brain disorder caused by a pathologically expanded CAG repeat (≥36) in the Huntingtin gene.^1^ Age at clinical onset is inversely correlated with CAG repeat length, explaining up to 84% of variability.^2^ The mean age at symptom onset is between 30 and 50 years (range 1.5–87).^3^ The term juvenile-onset Huntington disease (JHD) is arbitrarily defined for HD patients with symptom onset <21 years, which is seen in approximately 0.5%–5% of patients with HD.^4,5^ Clinical differences exist between JHD patients with disease onset in childhood (cJHD; onset ≤10 years) and in adolescence (aJHD; onset between 11 and 20 years).^6^ cJHD, mostly associated with CAG repeats ≥80, represents a different and more aggressive HD subtype.^7^

Over the years, various retrospective JHD case reports and series aided our understanding of this subtype of HD.^6^ Patients with JHD often present with a combination of neurocognitive impairments (decline in attention, memory, or school performance); psychiatric (e.g., irritability and depression) and behavioral disturbances; early onset of gait, speech, and swallowing disturbances; and a hypokinetic-rigid syndrome. In addition, other HD symptoms such as sleep disturbances, epileptic seizures, pain, and weight loss are commonly described. Furthermore, systemic disease manifestations in cardiovascular, respiratory, and gastrointestinal domains are frequently observed in HD and in some instances correlate with age at disease onset or CAG repeat length.^8-11^ The comparison of clinical characteristics between patients with cJHD, aJHD, and adult-onset HD (AHD) becomes more relevant when considering pathophysiologic differences between these age at onset–defined HD (AO-HD) subtypes.^12^ However, comparative studies between patients with JHD subtypes and AHD are rarely performed. One such study revealed faster progression of motor symptoms and shorter survival of patients with cJHD compared with those with aJHD and AHD.^7^ Other studies highlighted differences between patients with JHD and AHD regarding neurocognitive and psychiatric changes at the onset of disease^13,14^ and epilepsy^15^; however, they did not differentiate between childhood-onset and adolescent-onset of disease. To address JHD subtype differences and to ensure participation of patients with JHD in future interventional studies, quantification of expected clinical differences between AO-HD subtypes is essential.

The major limitation of studying the JHD phenotype is its low prevalence. To allocate as much JHD cases as possible, we started in 2020 a national Dutch registry for juvenile-onset HD patients (HD-JUNIOR). By the combined use of HD-JUNIOR and the international Enroll-HD platform,^16^ the objective of this study was to describe and compare JHD subtypes with AHD in the occurrence and severity of clinical characteristics at onset and during the disease course. Compared with AHD, we hypothesize that JHD subtypes will have a higher proportion of psychiatric and neurocognitive disease characteristics at onset and a higher occurrence of behavioral changes, epilepsy, and pain during the course of the disease. We also hypothesize that cJHD will have more severe motor disease characteristics related to hypokinetic-rigid syndrome, dystonia, and dysarthria and less severe chorea compared with aJHD and AHD.

## Methods

### Study Design and Population

To analyze JHD patient data from as many patients as possible and to allow for the comparison of JHD with typical disease onset in adulthood, data from 2 (J) HD datasets were used: the HD-JUNIOR and Enroll-HD registries. HD-JUNIOR was started in 2020 and retrospectively collects clinical data of both alive and deceased patients with JHD in the Netherlands (n = 28). Enroll-HD^16^ is an international (183 sites in 23 countries) prospective observational study since 2012 in which clinical data from (J) HD gene carriers—patients and controls—are gathered. Core Unified Huntington's Disease Rating Scale datasets were collected annually from all research participants as part of this multicenter longitudinal observational study. Data were monitored for quality and accuracy using a risk-based monitoring approach. Data were generously provided by the participants in the Enroll-HD study and made available by Cure Huntington's Disease Initiative (CHDI) Foundation, Inc. For this study, the 5th periodic dataset was used (PDS5; release 18-DEC-2020; n = 21,116 participants), including a specified dataset with deaggregated data for AO and enrollment younger than 17 years and CAG repeat length of 70 and higher.

**Figure 1:**
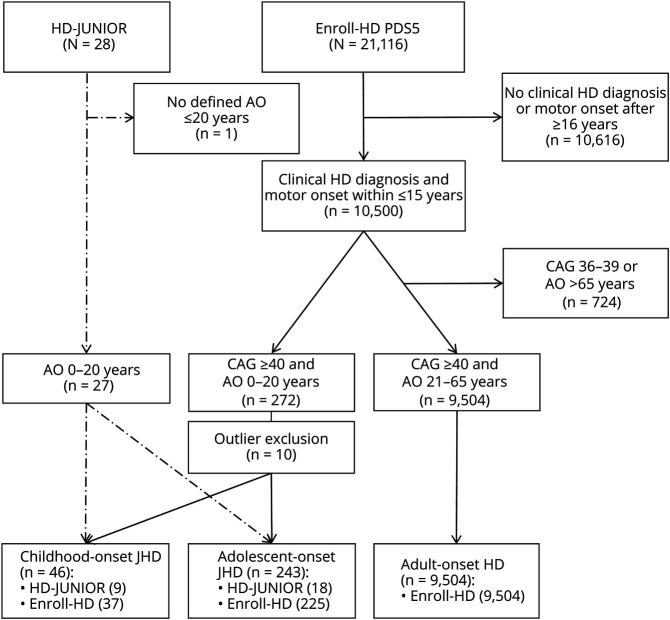
Participant Selection in HD-JUNIOR and Enroll-HD PDS5 The STROBE flow diagram illustrates the selection and stratification criteria for participants from the HD-JUNIOR (long dashed dot line) and Enroll-HD (solid line) datasets, detailing the number of patients who contributed data for 1 or more outcome measures in this study. Selected patients were stratified into 3 AO-HD subtypes: childhood-onset JHD, adolescent-onset JHD, and adult-onset HD. AO = age at onset; CAG = cytosine-adenine-guanine repeats in the Huntingtin gene; HD = Huntington disease; n = number of participants; PDS5 = periodic dataset 5; STROBE = Strengthening the Reporting of Observational Studies in Epidemiology.

Selection and stratification criteria for this study consisted of a clinical diagnosis of HD and AO-HD (AO-HD) subtypes (as defined below). Based on a higher suggested occurrence of psychiatric and neurocognitive disease characteristics in patients with JHD at onset,^6^ a JHD phenotype was primarily defined by any HD-related first symptom below 21 years of age and, subsequently, occurrence of motor symptoms within 15 years of first symptoms. Subsequently, patients with JHD were subdivided into childhood-onset JHD (cJHD: primary onset ≤10 years) and adolescent-onset JHD phenotype (aJHD: primary onset between 11 and 20 years). For the comparison of clinical characteristics in JHD subtypes with typical disease onset in adulthood, inclusion criteria for an AHD phenotype were any HD-related first symptom between age 21 and 65 years and a CAG repeat ≥40. In HD-JUNIOR, primary assessment of eligibility was performed by H.S.B and T.A.K. based on all available information in the medical records. In case of a questionable relationship of first symptom with JHD phenotype, S.T.B was consulted for confirmation or withdrawal of the patient with JHD in the registry. In Enroll-HD, participants were selected from the PDS5 by using the retrospective HD Clinical Characteristics (HDCC) questionnaire, including raters' estimate of age at first symptom onset, age at first motor symptom onset, and clinical HD diagnosis. Based on this selection, H.S.B., R.A.C.R, and S.T.B. then further analyzed clinical outliers of the JHD groups based on raters' confidence of AO estimation and time between age at onset and enrolment. Patients who we classified as outliers were removed from further analyses and are listed in eTable 1. See the Strengthening the Reporting of Observational Studies in Epidemiology (STROBE) flow diagram for the number of eligible AO-HD–defined patients in the HD-JUNIOR and Enroll-HD datasets (Figure 1). Five patients with aJHD were part of both datasets and therefore excluded from the HD-JUNIOR dataset in case of pooled analysis.

### Standard Protocol Approvals, Registrations, and Patient Consents

Local ethical approval for the conduct of assessments on human participants (Enroll-HD, NCT01574053) and use of pseudonymized clinical data (HD-JUNIOR) was provided by the Medical Research Ethical Committee of Leiden-The Hague-Delft (MREC-LDD). In addition, all participating sites in Enroll-HD were required to obtain and maintain local ethical approval. Written informed consent was obtained from all participants (or guardians of participants) in the Enroll-HD registry and from all alive participants in the HD-JUNIOR registry. In the case of clinical data from deceased patients with JHD in the HD-JUNIOR registry, the MREC-LDD determined that consent was not required and pseudonymized data were shared by the last treating physician.

### Outcome Variables

Our aim was to analyze and compare clinical characteristics at disease onset and throughout the disease course across the 3 key neurologic domains in HD: (1) motor, (2) neurocognitive, and (3) psychiatric, as well as (4) other domains. For the cross-sectional analysis of the prevalence of HD clinical characteristics at onset and occurrence of psychiatric characteristics during the disease course, retrospective data of both datasets were pooled (HD-JUNIOR: patient/caretaker answer retrieved from medical records; Enroll-HD: patient answer in the HDCC questionnaire) because of comparable outcome and assessment method. For the analysis of disease characteristics at onset, we defined 3 outcome variables:(1) (mixed) motor onset, (2) (mixed) neurocognitive onset, and (3) (mixed) psychiatric onset. For the occurrence of psychiatric disease characteristics, we specified 6 subclusters of which comparable data were available in both datasets: (1) irritability, (2) violent/aggressive behavior, (3) depression, (4) apathy, (5) perseverative and obsessive-compulsive behavior, and (6) psychosis. For both outcomes, patients were omitted from analyses in case of missing data.

To perform a cross-sectional analysis of the occurrence and severity of motor characteristics in AO-HD subtypes across its disease course, we defined subdomains of motor function and, because of differences in assessment methods, separated the results from the 2 datasets. For the HD-JUNIOR cohort, we used retrospective data from both the cJHD and aJHD patient groups (as reported by patients/caregivers and based on neurologic examinations) to examine the proportional occurrence of motor signs and symptoms over the course of the disease, categorized into the following subdomains: (1) speech and swallowing, (2) walking and balance, (3) parkinsonism, (4) excessive movement, (5) oculomotor dysfunction, and (6) other motor features. Prospective data from the UHDRS-Total Motor Score (UHDRS-TMS) in the Enroll-HD dataset were used to assess and compare the severity of motor symptoms across the 3 age-of-onset (AO-HD) subtypes. To use the most optimal sample size for the cJHD subtype and ensure clinical relevance with respect to disease progression, the cross-sectional analysis was performed on data of participants who were 6–10 years post-disease onset when participating in the study. For participants with multiple assessments within this time window, the mean score was calculated and used for analysis. Within the available items in the UHDRS-TMS, 6 outcome subclusters were defined based on their neuroanatomical and physiologic origins: (1) oculomotor (assessing horizontal and vertical ocular pursuit, saccade initiation, and velocity; maximum score = 24); (2) dysarthria (maximum score = 4); (3) chorea (involving the face, buco-oro-laryngeal region, trunk, and left and right upper and lower extremities; maximum score = 28); (4) dystonia (in the trunk and left and right upper and lower extremities; maximum score = 20); (5) parkinsonism (including body bradykinesia, left and right finger taps and rigidity; maximum score = 20); and (6) gait and balance (assessing gait, tandem walking, and retropulsion; maximum score = 12).

To assess the severity of neurocognitive disease characteristics during the disease course, we used neurocognitive measures (full, verbal, and performance IQ) based on neuropsychological assessments obtained from 7 patients with cJHD and 9 patients with aJHD from the HD-JUNIOR dataset. We decided not to perform a comparative analysis between age-of-onset (AO-HD) subtypes for neurocognitive measures from the Enroll-HD dataset because of insufficient data for the cJHD subtype.

In the *other* domain, we performed cross-sectional analysis on the occurrence of epilepsy and pain during the disease course. Regarding epilepsy, in HD-JUNIOR, epileptic seizures were recorded in case it was mentioned by the patient, caretaker, or medical expert and confirmatory EEG data or summary were available. In the Enroll-HD dataset, we used the comorbidity information to select all patients with an ICD-10 registration “G40”: epilepsy and recurrent seizures. For both datasets, the occurrence of epilepsy was recorded as *not present* in case of no specified data on the outcome. The occurrence of epilepsy was pooled between datasets because the assessment method for epilepsy in both relate to a clinical diagnosis of epileptic seizures. Regarding the assessment of pain, we specified the occurrence of pain in case it was mentioned by the patient or caretaker in the medical file. The occurrence of pain was recorded as *not present* in case of no specified data on the outcome. Different from the retrospective assessment of pain in the HD-JUNIOR dataset, we used the prospective Short Form health survey (SF12) at baseline using the Enroll-HD dataset to assess pain interference during daily activities in the past week and to compare it between AO-HD subtypes.^17^

### Statistics

All analyses were performed using R Statistical Software (v4.3.2; R Core Team^18^ 2020). The tidyverse (v2.0.0; 2019) package was used for statistical analyses.^19^

Pairwise comparisons for proportions (Z-test) were performed to compare AO-HD subtypes of the pooled datasets on the occurrence (yes/no) of (1) motor, neurocognitive, or psychiatric disease features at onset; (2) specified psychiatric disease characteristics during the disease course; (3) epileptic seizures during the disease course; and (4) pain interference during the disease course. Adjustment of *p* values for 3x multiple testing (1: cJHD vs aJHD, 2: cJHD vs AHD and 3: aJHD vs AHD) was performed using the Holm method. *p* Values < 0.05 (2-tailed) were considered statistically significant.

Kruskal-Wallis test was conducted to compare AO-HD subtypes of Enroll-HD on the severity of specified motor disease characteristics. Pairwise Wilcoxon rank sum tests with adjustment for 3x multiple testing using the Holm method was used for multiple comparisons. *p* Values < 0.05 (2-tailed) were considered statistically significant.

### Data Availability

Enroll-HD anonymized data are available upon request through the CHDI Foundation, Inc. For additional information regarding HD-JUNIOR data for research purposes, the principal investigator S.T. de Bot, MD, PhD may be contacted.

## Results

### Demographic Characteristics of the AO-HD Subtypes Per Dataset

The number of included patients of the HD-JUNIOR and Enroll-HD datasets and stratified by AO-HD subtype is provided in Table 1. No clinically meaningful difference was observed between the JHD samples of the 2 different datasets regarding CAG repeat length and age at primary onset. Owing to missing values and made selections (as described in Methods), the number of participants for the specified outcome measures may slightly differ from the number given in Table 1 and is therefore explicitly stated per outcome measure.

**Table 1:** Patient Sample Characteristics per AO-HD Subtype and Dataset

	Childhood-onset JHD		Adolescent-onset JHD		Adult-onset HD
	HD-JUNIOR (n = 9)	Enroll-HD (n = 37)	HD-JUNIOR (n = 18)	Enroll-HD (n = 225)	Enroll-HD (n = 9,504)
Age at primary onset, mean ± SD	6.70 ± 2.10	6.50 ± 2.60	16.60 ± 2.40	16.80 ± 2.50	44.70 ± 10.40
Age at enrollment, mean ± SD (range)	17.90 ± 3.00 (15.00–23.00)	16.60 ± 6.50 (7.00–29.00)	28.00 ± 5.50 (20.00–39.00)	26.80 ± 5.80 (13.00–47.00)	51.70 ± 11.40 (19.00–92.00)
Years between primary and motor onset mean ± SD (range)	1.00 ± 2.10 (0.00–6.00)	2.90 ± 3.60 (0.00–12.00)	0.90 ± 1.30 (0.00–4.00)	3.20 ± 4.00 (0.00–15.00)	1.20 ± 2.50 (0.00–15.00)
Years between primary onset and enrollment mean ± SD (range)	11.30 ± 3.00 (7.00–17.00)	10.10 ± 5.50 (1.00–17.00)	11.40 ± 5.30 (4.00–23.00)	10.00 ± 5.40 (0.00–19.00)	7.00 ± 5.60 (−7.00—47.00)
Follow-up time since enrollment in y mean ± SD (range)	n/a	1.50 ± 1.70 (0.00–6.20)	n/a	1.80 ± 1.70 (0–7.10)	1.90 ± 1.70 (0–7.60)
Sex					
M/F %	56.00/44.00	54.00/46.00	61.00/39.00	51.00/49.00	49.00/51.00
CAG repeat mean ± SD (range)	77.00 ± 9.00 (66.00–92.00)	75.00 ± 17.00 (48.00–110.00)	58.00 ± 6.00 (49.00–68.00)	57.00 ± 8.00 (41.00–81.00)	44.00 ± 3.00 (40.00–65.00)
Inheritance paternal/maternal %	89.00/11.00	80.00/20.00	76.00/24.00	67.00/32.00	48.00/52.00

Abbreviations: CAG repeat = cytosine-adenine-guanine repeats in the Huntingtin gene; F = female; M = male; n/a = not applicable; OC = outlier criterion; SC = selection criterium.

AO-HD sample characteristics related to age at primary symptom onset (SC), age at enrollment in the dataset (for HD-JUNIOR, this corresponds to age at death if medical records were obtained posthumously), years between primary symptom and first motor symptom onset (SC), years between primary symptom onset and enrollment in the dataset (OC for Enroll-HD), and follow-up time since enrollment (not applicable for HD-JUNIOR, because data are collected retrospectively). Additional characteristics include sex, CAG repeat length (SC for patients with adult-onset HD in Enroll-HD), and inheritance.

### Disease Characteristics at Onset

**Figure 2:**
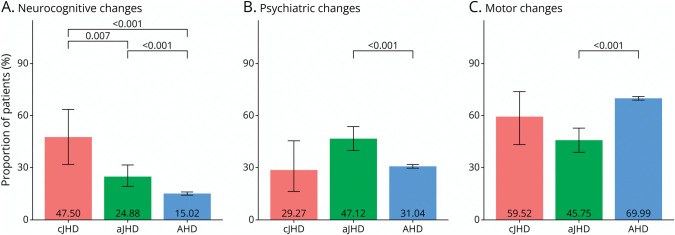
Prevalence and Comparison of HD Disease Characteristics at Onset Proportion of patients, 95% CI, and significant *p* values* per AO-HD subtype (cJHD = red bar; aJHD = green bar; AHD = blue bar) having (A) neurocognitive, (B) psychiatric, and (C) motor changes, whether combined or isolated, at disease onset (pooled datasets). For disease duration and follow-up time, refer to Table 1, and for stratified outcomes per dataset, see eTable 2. *p Values <0.050 are considered statistically significant and were adjusted for 3 comparisons (cJHD vs aJHD, cJHD vs AHD, and aJHD vs AHD) using the Holm method (inflated *p* values). AHD = adult-onset Huntington disease; aJHD = adolescent-onset (juvenile) Huntington disease; AO-HD = AO-defined HD subtype; cJHD = childhood-onset (juvenile) Huntington disease.

The prevalence of disease characteristics at onset were analyzed and compared between AO-HD subtypes in pooled data from the 2 datasets (Figure 2). Stratified counts and proportions per dataset are listed in eTable 2.

In the cJHD subtype, 19 (47.50%) patients (n = 40, 95% CI 31.80–63.70) presented with a neurocognitive phenotype, significantly more often than 52 (24.88%) patients with aJHD (n = 209, 95% CI 19.30–31.40, *p =* 0.007) and 1,228 (15.02%) patients with AHD (n = 8,177, 95% CI 14.30–15.80, *p* < 0 .001) (Figure 2A). The prevalence of neurocognitive signs at onset in patients with aJHD was also significantly higher compared with patients with AHD (*p* < 0.001). Specified initial disease characteristics were further analyzed in patients with JHD of the HD-JUNIOR dataset (eTable 3; cJHD n = 9, aJHD n = 17) because these types of data were not available in the Enroll-HD dataset. Initial neurocognitive changes were most often encountered as learning difficulties (cJHD 3 of 9; aJHD 2 of 18) and attention deficit (cJHD 2 of 9; aJHD 1 of 18). Furthermore, 5 of 9 patients with cJHD presented with developmental regression and 1 of 9 with developmental delay. Of these 6 of 9 cJHD patients with changes in development, 4 were related to initial changes in motor development (fine motor skills and walking pattern) and 2 to initial changes in neurocognitive development (repeating class and need for special education).

In the aJHD subtype, 98 (47.12%) patients (n = 208, 95% CI 40.20–54.10) presented with psychiatric signs and complaints, significantly more often when compared with 2,630 (31.04%) patients with AHD (n = 8,472, 95% CI 30.10–32.00, *p* < 0 .001) (Figure 2B). Furthermore, the aJHD subtype had in 97 (45.75%) patients (n = 212, 95% CI 39.00–52.70) motor signs and symptoms at onset (Figure 2C), significantly less often when compared with 6,040 (69.99%) patients with AHD (n = 8,630, 95% CI 69.00–71.00, *p* < 0.001). The most prevalent initial psychiatric change of patients with aJHD within the HD-JUNIOR dataset (eTable 3) was irritable and aggressive behavior (7 of 16 patients), which was not observed in 9 patients with cJHD at onset.

### Occurrence and Severity of Disease Characteristics During the Disease Course

In the next part, the occurrence and severity of changes during the course of the disease will be discussed within the 3 main HD domains—psychiatric, motor, and neurocognitive—and in the *other* domain.

### Psychiatric Disease Characteristics During the Disease Course

**Figure 3:**
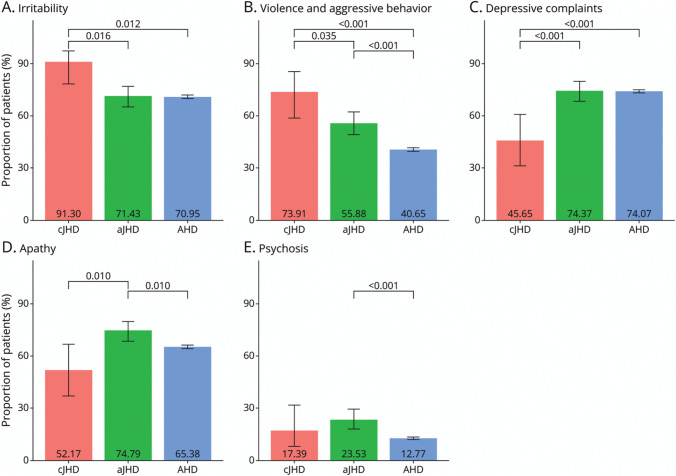
Occurrence and Comparison of Psychiatric HD Disease Characteristics During the Disease Course Proportion of patients, 95% CI, and significant *p* values* per AO-HD subtype (cJHD = red bar; aJHD = green bar; AHD = blue bar) experiencing (A) irritability, (B) violent and aggressive behavior, (C) depressive complaints, (D) apathy, and (E) psychosis as psychiatric disease characteristics (pooled datasets) during the course of the disease. For disease duration and follow-up time, refer to Table 1, and for stratified outcomes per dataset, see eTable 4. *p Values <0.05 are considered statistically significant and were adjusted for 3 comparisons (cJHD vs aJHD, cJHD vs AHD, and aJHD vs AHD) using the Holm method (inflated *p* values). AHD = adult-onset Huntington disease; aJHD = adolescent-onset (juvenile) Huntington disease; AO-HD = AO-defined HD subtype; cJHD = childhood-onset (juvenile) Huntington disease.

The occurrence of psychiatric disease characteristics during the disease course was analyzed and compared between AO-HD subtypes in pooled data from the 2 datasets (Figure 3; for disease duration, see Table 1). The stratified numbers of patients and proportions per dataset are listed in eTable 4.

Ever since primary onset, irritability occurred in 42 (91.30%) patients with cJHD (n = 46, 95% CI 78.30–97.20) and violence and aggressive behavior in 34 (73.91%) patients with cJHD (n = 46, 95% CI 58.60–85.20), significantly more often when compared with 170 (71.43%) (n = 238, 95% CI 65.20–77.00, *p =* 0.016) and 133 (55.88%) patients with aJHD (n = 238, 95% CI 49.30–62.30, *p =* 0.035) and 6,741 (70.95%) (n = 9,501, 95% CI 70.00–71.90, *p =* 0.012) and 3,863 (40.65%) patients with AHD (n = 9,502, 95% CI 39.70–41.70, *p =* <0.001) for irritability and aggressive behavior, respectively (Figure 3, A and B). By contrast, depressive complaints occurred in 21 (45.65%) patients with cJHD (n = 46, 95% CI 31.20–60.80), significantly less often compared with 177 (74.37%) patients with aJHD (n = 238, 95% CI 68.20–79.70, *p* < 0.001) and 7,039 (74.07%) with AHD (n = 9,503, 95% CI 73.20%–74.90%, *p* < 0.001) (Figure 3C).

Also, in patients with aJHD, violence and aggressive behavior occurred more often than in patients with AHD (*p* = <0.001). Furthermore, apathy occurred in 178 (74.79%) patients with aJHD (n = 238, 95% CI 68.70–80.10), significantly more often when compared with 24 (52.17%) patients with cJHD (n = 46, 95% CI 37.10–66.90, *p =* 0.010) and 6,212 (65.38%) with AHD (n = 9,502, 95% CI 64.40–66.30, *p =* 0.010) (Figure 3D). In addition, psychosis occurred in 56 (23.53%) patients with aJHD (n = 238, 95% CI 18.40–29.50), which was significantly more often compared with 1,213 (12.77%) patients with AHD (n = 9,502, 95% CI 12.10–13.50, *p* < 0.001) (Figure 3E). Perseverative and obsessive behavior occurred in 155 (65.13%) patients with aJHD (n = 238, 95% CI 58.70–71.17), 32 (69.57%) patients with cJHD (n = 46, 95% CI 54.25–82.26), and 5,419 (57.03%) patients with AHD (n = 9,502, 95% CI 56.03–58.03). No statistically significant differences (*p* > 0.05) were observed between patients with cJHD, aJHD, and AHD in the occurrence of perseverative and obsessive behavior.

### Motor Disease Characteristics During the Disease Course

To study specified motor disease characteristics during the disease course, the occurrence was analyzed in JHD subtypes of the HD-JUNIOR dataset (Table 2). In addition, the severity 6–10 years after onset was analyzed and compared in AO-HD subtypes based on UHDRS-TMS items of the Enroll-HD dataset (Table 3).

**Table 2:** Occurrence of Motor HD Disease Characteristics During the Disease Course of the HD-JUNIOR Dataset

	cJHD, (%)	aJHD, (%)
Speech and swallowing		
Anamnesis		
Difficulty speech	7/9 (77.78)	17/18 (94.44)
Difficulty swallowing	8/9 (88.89)	16/18 (88.89)
Sialorrhea	3/9 (33.33)	0/18 (0.00)
Neurological examination		
Dysarthria	9/9 (100)	14/17 (82.35)
Aphasia	1/9 (11.11)	1/17 (5.88)
Walking and balance		
Anamnesis		
Difficulty walking	8/9 (88.89)	14/18 (77.78)
Difficulty keeping balance	6/9 (66.67)	11/18 (61.11)
Neurological examination		
Gait disorder	9/9 (100)	13/17 (76.47)
Parkinsonian gait disorder	6/9 (66.67)	2/17 (11.76)
Dystonic gait disorder	2/9 (22.22)	3/17 (17.65)
Ataxic gait disorder	3/9 (33.33)	1/17 (5.88)
Spastic gait disorder	3/9 (33.33)	0/17 (0.00)
Balance disorder NOS	8/9 (88.89)	13/17 (76.47)
Parkinsonism		
Anamnesis		
Loss of fine motor skills	8/9 (88.89)	8/18 (44.44)
Stiffness	1/9 (11.11)	1/18 (5.56)
Neurological examination		
Rigidity	8/9 (88.89)	15/17 (88.24)
Hypokinesia	6/9 (66.67)	6/17 (35.29)
Bradykinesia	8/9 (88.89)	14/17 (82.35)
Micrography	2/9 (22.22)	0/17 (0.00)
Mask face	5/9 (55.56)	6/17 (35.29)
Excessive movement		
Anamnesis		
Excessive movement extremities	5/9 (55.56)	16/18 (88.89)
Excessive movement face	3/9 (33.33)	3/18 (16.67)
Tics (vocal or motor)	5/9 (55.56)	5/18 (27.78)
Neurological examination		
Chorea	5/9 (55.56)	16/17 (94.12)
Dystonia	6/9 (66.67)	10/17 (58.82)
Oral dyskinesia	4/9 (44.44)	1/17 (5.88)
Motor impersistence	3/9 (33.33)	9/17 (52.94)
Tics vocal	1/9 (11.11)	1/17 (5.88)
Tics motor	3/9 (33.33)	2/17 (11.76)
Tremor rest	1/9 (11.11)	0/17 (0.00)
Tremor action	2/9 (22.22)	2/17 (11.76)
Tremor intention	0/9 (0.00)	2/17 (11.76)
Tremor postural	0/9 (0.00)	1/17 (5.88)
Myoclonus	3/9 (33.33)	2/17 (11.76)
Oculomotor		
Neurological examination		
Ocular gaze abnormalities	5/9 (55.56)	9/17 (52.94)
Ocular saccade abnormalities	7/9 (77.78)	13/17 (76.47)
Other		
Neurological examination		
Dysdiadochokinesia	6/9 (66.67)	7/17 (41.18)
Dysmetria	2/9 (22.22)	1/17 (5.88)
Coordination disorder NOS	2/9 (22.22)	6/17 (35.29)
Hyperreflexia	7/9 (77.78)	4/17 (23.53)
Scoliosis	2/9 (22.22)	0/17 (0.00)
Apraxia	1/9 (11.11)	2/17 (11.76)

Abbreviations: aJHD = adolescent-onset (juvenile) Huntington disease; cJHD = childhood-onset (juvenile) Huntington disease; NOS = not otherwise specified.

Results are categorized by neuroanatomical and physiologic origin, as well as by source (complaints or signs reported by patients or caregivers during anamnesis, or symptoms identified during neurologic examination). The number of participants with each specified sign, symptom, or complaint is presented as a proportion of the total number of participants.

**Table 3:** Severity and Comparison of Motor Disease Characteristics 6–10 years After Primary Onset in the Enroll-HD Dataset

UHDRS-TMS subdomain	AO-HD subtype	*N*	Median score	IQR	Comparison	*p* Value
Dysarthria	cJHD	12	2.20	2.00	aJHD	**0.039**
	aJHD	93	1.00	1.00	AHD	**0.031**
	AHD	4,163	1.00	0.70	cJHD	**0.012**
Parkinsonism	cJHD	12	11.00	9.40	aJHD	0.077
	aJHD	93	8.50	6.80	AHD	**<0.001**
	AHD	4,158	6.00	4.70	cJHD	**0.008**
Dystonia	cJHD	12	6.50	8.20	aJHD	0.345
	aJHD	93	4.00	7.20	AHD	**0.015**
	AHD	4,161	2.00	5.20	cJHD	0.141
Oculomotor	cJHD	12	11.00	8.50	aJHD	0.493
	aJHD	93	9.30	9.20	AHD	0.146
	AHD	4,160	8.00	7.00	cJHD	0.263
Gait and balance	cJHD	12	3.50	8.00	aJHD	>0.999
	aJHD	93	3.70	4.30	AHD	>0.999
	AHD	4,159	4.00	3.80	cJHD	>0.999
Chorea	cJHD	12	5.00	4.20	aJHD	0.190
	aJHD	93	6.30	8.50	AHD	**<0.001**
	AHD	4,163	9.20	7.00	cJHD	**<0.001**
Total Motor Score	cJHD	12	45.80	40.40	aJHD	0.949
	aJHD	93	42.00	32.00	AHD	0.949
	AHD	4,148	38.00	25.00	cJHD	0.949

Abbreviations: AHD = adult-onset Huntington disease; aJHD = adolescent-onset (juvenile) Huntington disease; AO-HD = age at onset–defined HD subtype; cJHD = childhood-onset (juvenile) Huntington disease; IQR = interquartile range; UHDRS-TMS = Unified Huntington’s Disease Rating Scale—Total Motor Score.

UHDRS-TMS data from the Enroll-HD dataset were used to assess the severity of submotor domains 6–10 years after disease onset across the 3 AO-HD subtypes. Results are categorized by submotor domains, as detailed under Methods, and according to AO-HD subtype (cJHD, aJHD and AHD). The columns display the within-subtype median scores and IQR as measured 6–10 years after disease onset, along with between-group comparisons for each outcome measure. Row 1 compares cJHD with aJHD, row 2 compares aJHD with AHD, and row 3 compares AHD with cJHD. Statistical significance was adjusted for 3× multiple testing using the Holm method (inflated *p* values), *p* values <0.050 were considered statistically significant and are presented in bold.

Based on HD-JUNIOR, a high occurrence of symptoms related to speech and swallowing, walking and balance, parkinsonism, excessive movement, and oculomotor changes were eminent during the disease course in both JHD subtypes (Table 2). In the cJHD subtype, a gait disorder was observed in 6 of 9 patients specified as parkinsonian, in 3 of 9 as ataxic, in 3 of 9 as spastic, and in 2 of 9 as dystonic (overlap in specification was seen in 5 patients). In aJHD, although a gait disorder was mentioned in 13 of 18 patients, it was less often specified, with 3 patients having a dystonic gait disorder, 2 patients with parkinsonian gait disorder, and 1 patient with an ataxic gait disorder. The prototypical occurrence of chorea in HD was mentioned in 5 of 9 patients with cJHD and 16 of 17 patients with aJHD.

Based on UHDRS-TMS items in the Enroll-HD dataset (Table 3), a significantly higher median score in one or both JHD subtypes as compared with AHD was seen for dysarthria (AHD: n = 4,163, median score 1.00, interquartile range (IQR) 0.70; cJHD: n = 12, 2.20, 2.00, *p =* 0.039; aJHD: n = 93, 1.00, 1.00, *p* = 0.031), parkinsonism (AHD: n = 4,158, median score 6.00, IQR 4.70; cJHD: n = 12, 11.00,9.40, *p* = 0.008; aJHD: n = 93, 8.50, 6.80, *p* < 0.001), and dystonia (AHD: n = 4,161, median score 2.00, IQR 5.20; cJHD: n = 12, 6.50,8.20, *p* = 0.141; aJHD: n = 93, 4.00, 7.20, *p =* 0.015), as measured 6–10 years after onset. By contrast, a significantly lower median score in both JHD subtypes as compared with AHD was seen for chorea (AHD: n = 4,163, median score 9.20, IQR 7.00; cJHD: n = 12,5.00, 4.20, *p* = <0.001; aJHD: n = 93, 6.30, 9.50, *p* < 0.001). No statistically significant differences were observed between patients with cJHD, aJHD, and AHD in the median scores of UHDRS-TMS oculomotor and gait and balance items and the total motor score (Table 3).

### Neurocognitive Disease Characteristics During the Disease Course

The severity of neurocognitive disease characteristics during the disease course was analyzed in JHD subtypes of the HD-JUNIOR dataset (eTable 5). Patients with JHD often had a lower-than-average IQ as determined by the primary assessor (cJHD 4 of 7 vs aJHD 4 of 9). The mean total IQ score in the cJHD group was 80.8 ± 19.4 (years after onset: 3.7 ± 3.1) vs 75.8 ± 5 (years after onset: 5.4 ± 4.0) in the aJHD group. In general, performance IQ was lower than verbal IQ. This difference was largest in the aJHD group. An executive function disorder was specified in 1 patient with cJHD and 3 patients with aJHD, and an encoding deficit was specified in 4 patients with aJHD.

### Other Disease Characteristics During the Disease Course

The occurrence of epileptic seizures during the disease course was analyzed and compared between AO-HD subtypes in pooled data from the 2 datasets. Recurrent epileptic seizures occurred in 11 of 46 (23.91%) patients with cJHD (95% CI 13.10–39.10), significantly more often when compared with 15 of 237 (6.33%) patients with aJHD (95% CI 3.70–10.40, *p* < 0.001) and 852 of 9472 (0.90%) patients with AHD (95% CI 0.70–1.10, *p* < 0.001).

The occurrence of pain during the disease course was analyzed and compared between AO-HD subtypes for the separate datasets. Based on the HD-JUNIOR dataset, 5 of 9 patients with cJHD reported pain throughout their disease course. In patients with aJHD, this was 12 of 18 cases. Based on the Enroll-HD dataset, the occurrence of pain interference at baseline was 42.60% in aJHD patients (n = 129, 95% CI 33.70–51.50), which was significantly higher, in aJHD patients when compared with 11.80% in patients with cJHD (n = 17; 95% CI –2.50 to 26.10, *p* =0 .014) and 36.60% in patients with AHD (n = 4,262; 95% CI 36.20–37.00, *p =* 0.040).

## Discussion

This study identifies different disease characteristics at onset, as well as differences in the occurrence and severity of HD clinical characteristics over time in JHD subtypes compared with AHD. The cJHD population represents the extreme end of the HD spectrum, in which disease progression is known to be accelerated.^7^ By comparing a total of 46 patients with cJHD with those with aJHD and AHD, we confirm earlier reported findings such as (1) a high prevalence of neurocognitive abnormalities at disease onset,^7,13^ (2) more severe motor changes related to speech and parkinsonism,^6,7^ (3) less severe chorea,^6,7^ (4) neurocognitive changes reminiscent of the AHD phenotype,^20,21^ and (5) a higher occurrence of behavioral changes and lower depression complaints.^6,13^ Replication of these former findings confirms for their association with the cJHD phenotype in comparison with prototypical disease onset in adulthood.

The aJHD population is believed to be in closer resemblance with the AHD population compared with cJHD. By comparing 238 patients with aJHD with patients with cJHD and AHD, our study suggests alternative patterns in the aJHD population by (1) a higher prevalence of psychiatric abnormalities and a lower prevalence of motor changes at disease onset; (2) a higher occurrence of psychiatric changes related to psychosis and apathy during the disease course; (3) a higher occurrence of pain interference in daily life; and (4) motor changes related to speech, parkinsonism, and dystonia that are less severe than in cJHD but more severe than in AHD. These findings highlight that clinical characteristics in the aJHD population are not directly similar to those in the AHD population or its other counterpart, cJHD.

Our study is the first to report a higher occurrence of apathy and psychosis specifically in the aJHD population. This finding shows that the occurrence of psychosis in HD does not follow a linear relationship with AO or CAG repeat size. It is of interest that the predilection of this age group is also seen in the onset of idiopathic schizophrenia and might suggest similar risk factors in the onset of this phenotype.

Another interesting finding of our study is the higher occurrence of pain interference in daily life in patients with aJHD, when compared with patients with cJHD and AHD. A higher occurrence of pain has been linked to the JHD population before, but until now, a trend toward higher CAGs and therefore earlier onset of disease was observed.^22^ Furthermore, no comparison was made with AHD. Although a selection bias could influence our results (SF12 questionnaire in Enroll-HD is an optional part that is easily left out in case of a too high patient burden during annual visits), assessment of pain by using observational pain scales in addition to more extended self-reported pain scales in both patients with JHD and AHD could help clarifying the prevalence and origin of pain in different AO-HD subtypes.

We cannot exclude the possibility of bias influencing our study results. Information bias might have contributed to the lower estimates for depression in the cJHD population because depressive complaints are easily misrecognized in any childhood population.^23,24^ The fact that the HD-JUNIOR dataset uses unspecified medical data, however, does help minimize this risk. A selection bias may influence the cJHD population of Enroll-HD, because more severe cases are less likely to participate in prospective studies. Furthermore, we chose to omit missing cases from analyses. These patients might be different in certain respect from the patients who were included in our analyses, which would lead to some degree of selection bias. Another risk is a recall bias in the retrospectively collected data. Finally, using CAG repeat length, as used in previous studies, or using age at motor onset for the definition of JHD populations can be more accurate depending on the research question. In this study, we choose to define our groups by AO of any HD-related sign, rather than CAG repeat or age at motor onset. In our opinion, this definition relates better to patients presenting in clinical practice, prevents selection bias of patients with neurocognitive/psychiatric onset yet without a motor phenotype, and relates to a certain neurodevelopmental state that potentially influences disease characteristics.

The JHD population is a small heterogeneous group of patients that requires a tailored approach to what is known in HD research, as they represent the extreme end of the HD spectrum. We believe that future studies should include the structural comparison of AO-HD subtypes or different CAG repeat lengths in all types of (pre)clinical HD research. Furthermore, better identification of clinical characteristics such as developmental changes, gait abnormalities, and pain would help to understand their origin and therefore how to treat them. Finally, ongoing (international) collaborations are the only way forward in this very rare form of the disease. Pooling data from several JHD registries worldwide is an important next step in our understanding of JHD. To support these efforts, data from the Dutch HD-JUNIOR registry are available on request.

## Glossary

AHD: dult-onset Huntington disease
aJHD: adolescent-onset juvenile Huntington disease
AO: age at onset
AO-HD: age at onset–defined Huntington disease
CAG: cytosine-adenine-guanine
cJHD: childhood-onset juvenile Huntington disease
F: female
HD: Huntington disease
HDCC: HD Clinical Characteristics
IQR: interquartile range
JHD: juvenile-onset Huntington disease
M: male
MREC-LDD: Medical Research Ethical Committee of Leiden-Den Haag-Delft
N/A: not applicable
NOS: not otherwise specified
OC: outlier criterion
PDS5: 5th periodic dataset
SC: selection criterion
TMS: Total Motor Score
UHDRS: Unified Huntington’s Disease Rating Scale
